# Quality of Life in Patients Treated with Palliative Radiotherapy for Advanced Lung Cancer and Lung Metastases

**DOI:** 10.4021/wjon288w

**Published:** 2011-04-09

**Authors:** Kaitlin Koo, Liang Zeng, Florencia Jon, Emily Chen, Kristopher Dennis, Lori Holden, Liying Zhang, Amanda Caissie, Janet Nguyen, May Tsao, Elizabeth Barnes, Cyril Danjoux, Arjun Sahgal, Edward Chow

**Affiliations:** a Rapid Response Radiotherapy Program, Department of Radiation Oncology, Odette Cancer Center, Sunnybrook Health Sciences Center, University of Toronto, Toronto, Ontario, Canada

**Keywords:** QLQ-C15-PAL, QLQ-LC13, Quality of life, Advanced cancer, Radiotherapy, Lung, Metastases

## Abstract

**Background:**

The purpose of this study was to investigate quality of life (QOL) in patients receiving palliative radiotherapy (RT) for advanced lung cancer/lung metastases using the EORTC QLQ-LC13 and the EORTC QLQ-C15-PAL questionnaires.

**Methods:**

Patients who received palliative RT for lung metastases or advanced lung cancer between November 2007 and October 2010 completed the EORTC QLQ-LC13 and the QLQ-C15-PAL at baseline prior to RT, 1, 2, 4, 8 and 12 weeks post-treatment. The Wilcoxon Signed Rank test was used to compare QOL scores between baseline and each follow-up period.

**Results:**

Thirty-one patients with advanced lung disease were included in this study; 61% of participants were male and 39% were female. The median age was 69 years (range 38 - 85), and median KPS and PPS scores at baseline were both 70 (range 30 - 90). All patients received radiotherapy to the lung. None of the QLQ-LC13 scores significantly improved or deteriorated at any follow-up. Of the QLQ-C15-PAL scales, fatigue, pain, insomnia and physical functioning significantly improved at their respective follow-ups.

**Conclusions:**

This was the first study to use the EORTC QLQ-LC13 in conjunction with the EORTC QLQ-C15-PAL questionnaires. Future studies should continue to incorporate quality of life assessment tools specific to disease characteristics in advanced cancer patients.

## Introduction

Patients with advanced lung cancer or lung metastases (herein referred to as advanced lung disease) often present with thoracic symptoms such as hemoptysis, cough, chest pain, dysphagia and dyspnea. In the primary or non-metastatic setting, fewer than 15% of patients achieve long-term survival [1, 2]. Treatment intent is often palliative and health-related quality of life (QOL) becomes the primary treatment goal. Although patients’ self-reported QOL is a good indicator of overall well-being, it has infrequently been investigated in patients with advanced lung disease [3-9].

Palliative radiotherapy (RT) is effective in ameliorating symptoms experienced by patients with advanced lung disease [9-11] and has been shown to improve or at least preserve QOL [8]. There is limited literature that investigates QOL after treatment with palliative RT using validated lung symptom-specific tools.

The European Organization for Research and Treatment of Cancer (EORTC) addressed the need for standardized QOL tools by coordinating the development of both a generalized QOL questionnaire and disease-specific QOL questionnaires. The purpose of this study was to use the QLQ-C15-PAL and the QLQ-LC13 to investigate the effectiveness of RT in improving the QOL of patients receiving palliative radiotherapy for advanced lung disease.

## Methods

The Rapid Response Radiotherapy Program (RRRP) at the Odette Cancer Center, Sunnybrook Health Sciences Center, Toronto, Ontario, Canada, provides timely access to palliative RT for patients with advanced cancer. Patients referred to the RRRP between November 2007 and October 2010, receiving palliative radiotherapy for symptomatic advanced primary lung cancer or lung metastases were eligible for this study. Patients accrued to this study had evidence of malignancy, radiological evidence of primary lung cancer or metastases to the lung, spoke English and provided informed and written consent. Baseline information collected included age, gender, primary cancer site, Karnofsky Performance Status (KPS), and Palliative Performance Scale (PPS) score. Health-related QOL was assessed using the EORTC QLQ-C15-PAL and QLQ-LC13. Patients completed both questionnaires at baseline, prior to RT, and weeks 1, 2, 4, 8 and 12 from the start of RT. Baseline questionnaires were conducted in person and a trained research assistant completed subsequent telephone follow-up questionnaires. All research was conducted following approval from the Sunnybrook Health Science Center research ethics board.

### The EORTC QLQ-C15-PAL

The QLQ-C15-PAL is an abbreviated version of the commonly used EORTC QLQ-C30 [12]. The QLQ-C15-PAL consists of 15 questions: five items assessing physical and emotional functioning, four items assessing fatigue and pain, five item symptom scales (nausea/vomiting, dyspnea, insomnia, appetite loss, constipation) and one final question assessing overall QOL [13, 14].

### The EORTC QLQ-LC13

The EORTC QLQ-LC13 is a supplementary questionnaire for patients with lung cancer and includes questions assessing cough, hemoptysis, dyspnea, site-specific pain, treatment-related side effects (sore mouth, dysphasia, peripheral neuropathy and alopecia) and the efficacy of pain medications [15].

### Statistical analysis

The Wilcoxon Signed Rank test was used to determine changes in QLQ-C15-PAL and QLQ-LC13 scores between baseline and each follow-up visit (i.e., week 1, week 2, month 1, month 2, and month 3). All analyses were conducted by Statistical Analysis Software (SAS version 9.2 for Windows). Two-sided P-values of less than or equal to 0.05 were considered statistically significant.

## Results

### Patient demographics

A total of 31 patients were enrolled. Patient demographics are shown in Table 1. Sixty-one percent of participants were male and thirty-nine percent were female. Their median age was 69 years (range 38 - 85), and their median KPS and PPS scores at baseline were both 70 (ranges 30 - 90). At weeks 1, 2, 4, 8 and 12 weeks, 14 (45%), 14 (45%), 15 (48%), 12 (39%) and 9 (29%) patients completed follow-up questionnaires, respectively. The most common primary cancers were of the lung (80%) and colon (6%).

**Table 1:** Patients Demographics (N = 31)

Age (years)		
n	31	
Mean ± SD	68 ± 11	
Inter-quartiles	58 - 77	
Median (range)	69 (38 - 85)	
Karnofsky Performance Scale		
n	31	
Mean ± SD	67 ± 16	
Inter-quartiles	60 - 80	
Median (range)	70 (30 - 90)	
Palliative Performance Scale		
n	30	
Mean ± SD	65 ± 17	
Inter-quartiles	60 - 80	
Median (range)	70 (30 - 90)	
Gender		
Male	19	(61%)
Female	12	(39%)
Primary Cancer Site		
Lung	25	(80%)
GI-Colon	2	(6%)
GI-Rectum	1	(3%)
Breast	1	(3%)
Prostate	1	(3.23%)
Renal Cell	1	(3%)

### QLQ-C15-PAL score comparisons between baseline and each follow-up visit

**Figure 1:**
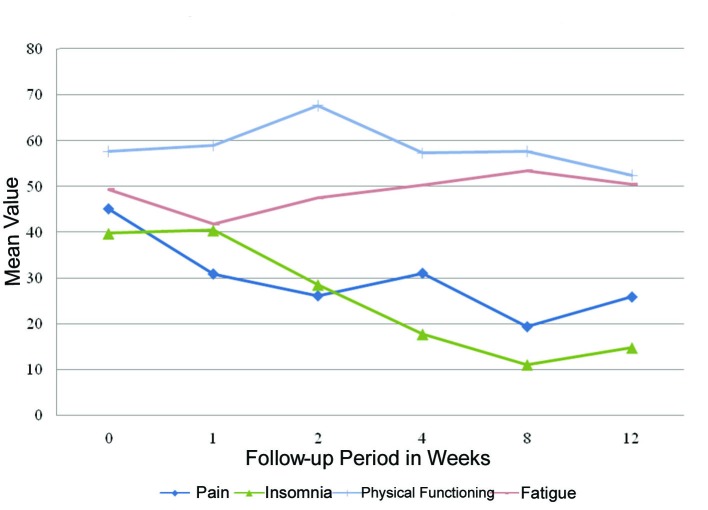
QLQ-C15-PAL scores with significant differences between follow-up and baseline.

Fatigue, pain, insomnia and physical functioning significantly improved from baseline at different time points during follow-up (Fig. 1). Fatigue significantly improved at week 1 when compared to baseline (P = 0.04). Pain significantly improved at week 2 (P = 0.03). Insomnia improved at months 1 (P = 0.008) and 2 (P = 0.05) while physical functioning improved at month 3 compared to baseline (P = 0.05). There were no significant differences or worsening of symptoms between any of the other items after receiving RT (Table 2).

**Table 2:** QLQ-C15-PAL Mean Score Value for Each Symptom Item at Baseline and Follow-up

Symptom Scales/Items	Data Collection Period in Weeks					
	0	1	2	4	8	12
**Pain**	45.16	30.95	**26.19**	31.11	19.44	25.93
Dyspnea	45.56	42.86	30.95	48.89	33.33	40.74
**Insomnia**	39.78	40.48	28.57	**17.78**	**11.11**	14.81
Appetite Loss	41.11	38.1	23.81	31.11	33.33	59.26
Constipation	30	28.57	21.43	24.44	16.67	40.74
Overall Quality of Life	51.67	54.76	50	56.67	52.78	55.56
**Physical Functioning**	57.7	59.05	67.69	57.33	57.78	**52.5**
**Fatigue**	49.46	**41.88**	47.62	50.37	53.54	50.62
Nausea/Vomiting	12.9	15.48	11.9	8.89	15.28	12.96
Emotional Functioning	74.14	75.6	85.12	82.22	75	64.81

QLQ-C15-PAL scores were compared between baseline and at each follow-up visit. Scores that show significant differences at the indicated follow-up period are bolded. Significant differences were calculated using Wilcoxon Signed Rank.

### QLQ-LC13 score comparisons between baseline and each follow-up visit

Coughing, hemoptysis, sore mouth, dysphagia, peripheral neuropathy, alopecia, pain in the chest, pain in the arm and dyspnea did not significantly change during any follow-up period (Table 3). The score for ‘other pain’ significantly improved at week 2 when compared to baseline (P = 0.02).

**Table 3:** QLQ-LC13 Mean Score Values for Each Symptom Item at Baseline and Follow-up

Symptom Scales/Items	Data Collection Period in Weeks					
	0	1	2	4	8	12
Coughing	49.46	42.86	45.24	42.22	36.11	25.93
Hemoptysis	16.13	16.67	5.13	4.44	8.33	11.11
Sore Mouth	6.45	7.69	4.76	6.67	8.33	7.41
Dysphagia	16.13	19.05	9.52	15.56	5.56	3.7
Peripheral Neuropathy	18.89	26.19	0	17.78	5.56	11.11
Alopecia	8.89	0	7.69	28.89	30.3	37.5
Pain in Chest	27.96	30.95	26.19	15.56	19.44	22.22
Pain in Arm	22.58	16.67	9.52	24.44	5.56	18.52
**Pain Other**	39.78	25.64	**12.82**	26.67	30.56	44.44
Dyspnea	30.95	33.33	16.67	30.56	26.39	36.51
Dsypnea When Rested	2.33	1.5	2.25	2	1.33	2.5
Dyspnea When Walked	2.33	2	2.75	2	1.67	3

QLQ-LC13 scores were compared between baseline and at each follow-up visit. Scores with a significant difference at the indicated follow-up period are bolded. Significant differences were calculated using Wilcoxon Signed Rank.

## Discussion

To our knowledge, this was the first study to use the EORTC QLQ-C15-PAL in conjunction with the EORTC QLQ-LC13 to investigate QOL in patients with advanced lung disease receiving palliative radiotherapy. The use of the QLQ-LC13 and the QLQ-C15-PAL are ideal in this setting due to their brevity, site- and patient-specific focus. In our study, we found ‘other pain’ was the only lung specific symptom that improved post-radiotherapy as assessed by the QLQ-LC13. However due to variability associated with the specific area of pain for this item, we cannot confidently include this within our findings. Pain, insomnia, physical functioning and fatigue, as measured by the QLQ-C15-PAL significantly improved at some period after treatment.

Fairchild et al. performed a meta-analysis investigating optimum palliative RT for advanced stage lung cancers [9], suggesting that both high and low RT dose schedules provided some level of improvement in thoracic symptoms secondary to lung cancer. As a reflection of the limited available literature, validated QOL data was compiled as part of the aforementioned analysis and identified as an area of research requiring further investigation. The authors indicated the need to include QOL as a meaningful endpoint when evaluating treatments for advanced lung disease; a sentiment echoed by other authors as well [7, 8, 13, 16-23].

Salvo et al. reviewed QOL assessment tools for patients receiving palliative radiotherapy for advanced lung cancer and lung metastases. This review encouraged investigators to include validated, specific QOL instruments such as the EORTC QLQ-LC13 or the FACT-L due to the specificity of these instruments in measuring lung-cancer specific symptoms [24].

Other studies assessing QOL in patients treated with palliative radiotherapy for advanced lung disease have used various other assessment tools such as the Spitzer QLQ Index, Hospital Anxiety and Depression Scale (HADS), Rotterdam Symptom Checklist (RSCL), study-designed QLQ questionnaires, Functional Assessment of Cancer Therapy-Lung/General (FACT-L/FACT-G), Lung Cancer Symptom Scale, and the EORTC QLQ-LC17 [24]. Salvo et al. suggested that use of validated, lung-specific tools (the FACT-L or EORTC QLQ-LC13) would allow for easier comparisons between trials and would also increase the internal validity of individual studies [24].

Several studies have used the QLQ-LC13 and the QLQ-C30 when assessing QOL in patients with lung cancer [8, 15, 19, 22, 23, 25]. Similarly, our study included patients with primary lung cancers and patients with lung metastases. The QLQ-C15-PAL was able to identify improvements in pain, insomnia, fatigue and physical functioning post-RT. These results are consistent with the findings of Hicsonmez, Bezjak and Langendijk [8, 20, 21]. Improvements in insomnia, fatigue and pain may result since each of these symptoms may contribute to the maintenance of the others, resulting in a significant adverse impact on QOL [26].

It is interesting to note that in our study, none of the lung specific symptoms assessed by the QLQ-LC13 significantly improved or deteriorated during any follow-up period. However, palliative radiotherapy may have played a stabilizing role. Hicsonmez et al. found that dyspnea significantly improved post-treatment and Langendijk et al. reported that palliative radiotherapy was effective in palliation of hemoptysis, chest pain and cough as assessed by the QLQ-C30 and QLQ-LC13 [8, 20]. Furthermore, Lutz et al. found statistically significant improvements in cough, hemoptysis and dyspnea using the Lung Cancer Symptom Scale [7]. Literature exists suggesting that symptoms experienced by this group actually worsened during and immediately following RT, and then returned to baseline levels [13, 14, 16, 19].

Due to the progression of the patient’s disease and limited prognosis, many patients were subsequently lost to follow-up. Our analysis of QOL and symptom trends may be more reflective of patients with better prognosis and may not truly represent this population. Radiation therapy for treatment of advanced lung disease is a well-tolerated therapeutic modality and preserves QOL. As the goals of treatment shift from survival to QOL, specific assessment tools such as the QLQ-LC13 should be incorporated in future clinical trials investigating patients with lung metastases or advanced lung cancer. More efforts should be directed towards investigating the outcomes of those patients who were lost to follow-up, as this was a common limitation expressed by similar studies.
